# Personalized anesthesia and precision medicine: a comprehensive review of genetic factors, artificial intelligence, and patient-specific factors

**DOI:** 10.3389/fmed.2024.1365524

**Published:** 2024-05-09

**Authors:** Shiyue Zeng, Qi Qing, Wei Xu, Simeng Yu, Mingzhi Zheng, Hongpei Tan, Junmin Peng, Jing Huang

**Affiliations:** ^1^Zhuzhou Clinical College, Jishou University, Jishou, China; ^2^Department of Anesthesiology, Zhuzhou Central Hospital, Zhuzhou, China; ^3^Department of Radiology, Third Xiangya Hospital, Central South University, Changsha, China

**Keywords:** personalized anesthesia, precision medicine, pharmacogenomics, biomarkers, monitoring technologies, machine learning, artificial intelligence

## Abstract

Precision medicine, characterized by the personalized integration of a patient’s genetic blueprint and clinical history, represents a dynamic paradigm in healthcare evolution. The emerging field of personalized anesthesia is at the intersection of genetics and anesthesiology, where anesthetic care will be tailored to an individual’s genetic make-up, comorbidities and patient-specific factors. Genomics and biomarkers can provide more accurate anesthetic protocols, while artificial intelligence can simplify anesthetic procedures and reduce anesthetic risks, and real-time monitoring tools can improve perioperative safety and efficacy. The aim of this paper is to present and summarize the applications of these related fields in anesthesiology by reviewing them, exploring the potential of advanced technologies in the implementation and development of personalized anesthesia, realizing the future integration of new technologies into clinical practice, and promoting multidisciplinary collaboration between anesthesiology and disciplines such as genomics and artificial intelligence.

## 1 Introduction

Precision medicine, a paradigm that individualizes healthcare by integrating a patient’s genetic blueprint and clinical history (1), is a rapidly evolving field that has demonstrated its potential across a broad range of biomedical areas and addressed significant public health challenges (2). This approach is particularly paramount in the realm of anesthetic management for enhancing patient safety and optimizing therapeutic efficacy. Additionally, individualized medicine plays a crucial role within precision medicine (3). Traditional anesthetic protocols, while effective for the majority, often overlook the vast inter-individual variability in drug responses and procedural risks. These differences can lead to unpredictable responses or toxic effects in some individuals or subgroups, ultimately impacting patient outcomes (4).

Personalized medicine opens up new horizons in the field (5). The advent of personalized anesthesia heralds a shift toward a nuanced framework where anesthetic regimens are sculpted around the patient’s genetic predispositions, existing comorbidities, and specific physiological parameters. This granular customization aims to mitigate perioperative complications, fine-tune pain management, bolster enhanced recovery after surgery (ERAS) protocols, and enhance patient satisfaction.

Recent strides in genomic sequencing, biomarker identification, and innovations in monitoring modalities have been pivotal in catapulting personalized anesthesia from conceptualization to clinical practice. Pharmacogenomics, which focuses on identifying genetic variations that affect the pharmacodynamics and pharmacokinetics of drugs, has shed light on genetic polymorphisms that modulate anesthetic sensitivity and susceptibility to complications, playing an important role in personalized medicine (6). Concurrently, the emergence of novel biomarkers and cutting-edge monitoring technology has refined the predictive accuracy of anesthetic outcomes, facilitating more strategic intraoperative planning.

This study searched the PubMed database using “personalized anesthesia,” “pharmacogenomics,” “biomarkers,” “machine learning,” and “artificial intelligence” as search terms for articles published between 2000 and 2024. The articles retrieved included clinical trials, randomized controlled trials, and reviews. These articles were then categorized according to the content of their abstracts. This review elucidates the intricacies of personalized anesthesia within the framework of precision medicine, emphasizing the influence of genetic variables, comorbid conditions, and individual patient factors on anesthetic administration. It canvasses the burgeoning domain of pharmacogenomics, explores the trajectory of biomarker and monitoring technology development, and scrutinizes the impediments and prospective evolution of personalized anesthesia. Furthermore, it considers the growing impact of artificial intelligence (AI) and machine learning as pivotal tools in the evolution of anesthetic precision.

## 2 Genetic factors

**FIGURE 1:**
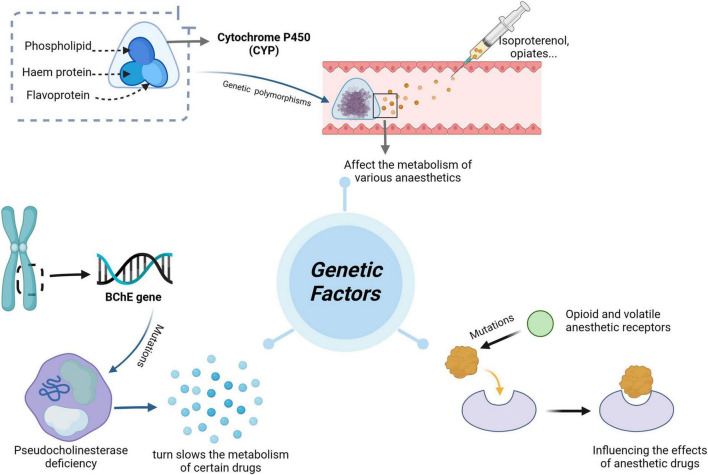
Genetic factors influence the effect of anesthesia.

Pharmacogenomics (PGx) is the discipline of predicting drug efficacy and toxicity at the genetic level (7), dedicated to elucidating the genetic variations that underlie the pharmacodynamics and pharmacokinetics of legacy drugs. This knowledge can guide the clinical selection of optimal therapeutic agents at the most appropriate dosage, improving drug efficacy, reducing or avoiding adverse effects, enhancing prognosis, and saving healthcare costs (8). This emerging field has evolved from merely identifying gene-drug pairs to realizing their clinical applications (9). Recent studies support the premise that adverse drug reactions (ADRs) can be prevented through PGx testing, highlighting this approach’s potential to improve drug safety and optimize therapeutic efficacy (10, 11). Figure 1 illustrates the relationship between genetic factors and the effects of anesthesia.

### 2.1 CYP450

Cytochrome P450 (CYP) is a collection of structurally and functionally related isoenzymes belonging to the group of B cytochromes. This group includes flavoprotein (NADPH cytochrome C reductase), hemoglobin (P450), and phospholipids (phosphatidylcholine). These enzymes are found mainly in the liver (4), but also in the lungs, kidneys, brain, and, to a lesser extent, in the gastrointestinal tract, skin, and placental tissue. CYP proteins, particularly those of the CYP1, CYP2, and CYP3 families, are major contributors to drug metabolism in humans (12). Their function and expression are regulated by variables such as sex, age, and disease state (13). CYP450 is the first step in the metabolism of almost 80% of drugs (14). Table 1 summarizes the genotypes discussed in this chapter and their impact on drugs used in anesthetic practice.

**TABLE 1:** Summary of anesthetic drugs and genotypes affecting their pharmacological effects.

Drug class	Drug	Gene	Major variants	Effect on drug pharmacokinetics/pharmacodynamics and clinical outcomes
Volatile anesthetics	Desflurane Isoflurane Sevoflurane	CYP2E1	CYP2EI*2	
			CYP2EI*5	Approximately 70% of patients with halogenated anesthetic halothane-induced hepatic inflammation patients develop autoantibodies specifically against *CYP2E1* (32)
Intravenous anesthetics	Propofol	CYP2B6	CYP2B6*4	Reduced dose requirements for isoproterenol under general anesthesia in patients carrying the CYP2B6*6 T allele (33)
			CYP2B6*5	
			CYP2B6*6	
		CYP2C9	CYP2C9*2 CYP2C9*3	Variants of CYP2C9 (*2, *3) associated with increased risk of hemorrhage (34, 35)
	Ketamine	CYP2B6	CYP2B6*1 CYP2B6*6	CYP2B6*6 diplotype may have decreased clearance; Safety: increased risk of adverse effects
	Midazolam	CYP3A4	CYP3A4*22	Decreased enzyme activity; increased midazolam plasma concentrations; increased risk of adverse events (16, 27, 36)
		CYP3A5	CYP3A5*1	Increased metabolism of midazolam (17)
Opioid analgetics	Codeine	CYP2D6	CYP2D6*10	As the number of CYP2D6*10 alleles increases, codeine-forming morphine Cmax and AUC of codeine-forming morphine decreased (37)
	Oxycodone		CYP2D6*4/*4 CYP2D6*4/*6	Associated with nausea and vomiting following oxycodone administration (38)
	Tramadol		CYP2D6 NM CYP2D6 IM	Associated with enhanced analgesic efficacy of opioids in the treatment of postoperative pain (39)
	hydrocodone		CYP2D6*4/*4	Adverse events, including nausea and vomiting, were observed in subjects with the CYP2D6*4/*4 genotype. including nausea and vomiting (38)
	Fentanyl	CYP3A4 CYP3A5	CYP3A4*1G	Patients carrying the *1G allele require higher doses of fentanyl to achieve adequate analgesia (40)
Antiemetics	Tropisetron	CYP2D6	Ultrarapid phenotype	Reduced antiemetic effect of ondansetron and tropisetron in postoperative or chemotherapy-induced nausea and vomiting observed in ultra-rapid metabolizers of *CYP2D6* (41, 42)
	Ondansetron	CYP1A2 CYP2D6 CYP3A4		

Inhalational anesthetics are among the most commonly used general anesthetic agents in clinical anesthesia and have general anesthetic, analgesic, sedative, and amnestic effects. Between 20 and 50% of halothane, 2% of sevoflurane, less than 1% of isoflurane, and 0.1% of desflurane are biotransformed in the liver (15). Metabolism occurs in the liver and kidney via microsomal *CYP2E1*. Inhalational anesthetics enter the body and, due to their high lipophilicity, are rapidly absorbed into the circulation and distributed to the tissues; they are almost exclusively eliminated by the lungs. Therefore, their effects do not depend on common polymorphisms in genes encoding metabolic enzymes or drug transporter proteins (16, 17). Hepatotoxicity of halothane has been frequently reported (18). Hepatotoxicity of desflurane, sevoflurane, and isoflurane has also been occasionally reported (19, 20).

Propofol is the most commonly used parenteral anesthetic with sedative-hypnotic, anxiolytic, anticonvulsant, anti-inflammatory, antiemetic, antioxidant, and possibly neuroprotective effects. Differential responses to propofol may be due to polymorphisms in the gene encoding the metabolic enzyme CYP2B6 (7). Up to 70% of propofol binds to glucuronide via UGT1A9, while the remaining 30% of the drug is first hydroxylated via *CYP2B6* (17). Iohom et al. (21) found that interpatient variability in response was associated with the presence of *CYP2B6* variants (R487C, K262R, and Q172), but *GABRE* variants (mRNA358G/T, 20118C/T, 20326C/T, and 20502A/T) were not statistically significantly associated.

Ketamine, a potent analgesic, increases heart rate, blood pressure, and cardiac output. It acts mainly at NMDA receptors as a non-competitive blocker. Ketamine is metabolized primarily by two cytochrome P450 enzymes, CYP2B6 and CYP3A4, and is subsequently glucuronidated and excreted by the kidneys (22, 23). Li et al. (24) found that the CYP2B6*6 allele was associated with a significant reduction in steady-state ketamine plasma clearance in chronic pain patients.

Midazolam, a common benzodiazepine sedative-hypnotic, exhibits sedative-hypnotic, anxiolytic, anticonvulsant, myorelaxant, and amnesic properties. It is primarily metabolized by CYP3A4 and CYP3A5, and its metabolites bind to glucuronide (25). The CYP3A4*22 variant is associated with reduced enzyme function (26). POR is an important component of the CYP enzyme system, and POR28 is a common variant. One study found a 45% lower metabolism of midazolam in patients with the POR*28 variant compared to those with the POR*1/*1 genotype among *CYP3A5* expressors (27).

Opioids, the most commonly used analgesics, are metabolized by CYP2B6, CYP2D6, CYP3A4, and CYP3A5. Tramadol is metabolized in the liver by CYP2D6 into its pharmacologically active metabolite, O-desmethyltramadol. Fentanyl is metabolized in the liver by CYP3A4 and CYP3A5 into desmethylfentanyl. CYP3A5*1 is the only functional allele known to enhance fentanyl metabolism (16). Codeine is metabolized in the liver into morphine by CYP2D6, and patients with poor CYP2D6 metabolism exhibit very low morphine plasma concentrations after codeine administration (28). The majority of oxycodone is metabolized by CYP3A4 into its inactive metabolite, noroxycodone, while a minority is metabolized by CYP2D6 into its active metabolite, oxymorphone (28, 29). Metabolism varies among *CYP2D6* genotypes, and the analgesic effect of oxycodone is diminished in individuals with poor metabolism compared to those with extensive metabolism. Alternative medications should not be metabolized by CYP2D6 and therefore should not contain oxycodone, tramadol, or codeine, which are metabolized by CYP2D6 (30).

Ondansetron and tropisetron, commonly used to prevent post-operative nausea and vomiting (PONV), are 5-HT3 receptor antagonists. Ondansetron is metabolized in the liver by CYP1A2, CYP2D6, and CYP3A4, while tropisetron is primarily metabolized by CYP2D6. Ultra-fast *CYP2D6* metabolizers of ondansetron experience a higher incidence of vomiting and reduced antiemetic efficacy. It is recommended that dosing be consistent for individuals with moderate and poor CYP2D6 metabolizing phenotypes. For ultra-fast metabolizers, antiemetics that do not rely on CYP2D6 substrates are advised (31).

### 2.2 Pseudocholinesterase

Pseudocholinesterase, or butyrylcholinesterase, is an esterase that is expressed throughout the body and encoded by the *BChE* gene on chromosome 3q26 (43). Mutations in this gene can lead to pseudocholinesterase deficiency, which in turn slows the metabolism of certain drugs, resulting in delayed metabolic conditions (44). Changes in pseudocholinesterase activity can cause prolonged apnea. Among these changes, the A variant (*209A* > *G, Asp70Gly*) and the K variant (*1615G* > *A, Ala539Thr*) are the most common (45). The elimination of ester-type anesthetics such as bupivacaine and procaine depends on plasma butyrylcholinesterase activity. Case studies have highlighted the long-term effects of epidural injections of chloroprocaine in patients with abnormal pseudocholinesterase activity (46, 47). Additionally, individuals with this deficiency may experience long-term paralysis after the administration of succinylcholine due to impaired drug metabolism (48, 49).

### 2.3 Receptor polymorphisms

With regard to receptor polymorphisms, opioid and volatile anesthetic receptors (e.g., mu opioid receptor (OPRM1) and gamma-aminobutyric acid type A (GABAA) receptors) have been shown to result in different patient responses to anesthetics. Opioid receptors are widely expressed in the central nervous system and peripheral tissues, and the μ-opioid receptor encoded by OPRM1 is a major binding site for opioids (50). More than 200 variant alleles of this gene have been identified. Genetic differences arising from variations in these genes are a major source of variability in opioid response (51). The *OPRM1 118 A* > *G* variant can alter μ-opioid receptor (MOR) signaling in the brain (52). Studies have shown that individuals with at least one *OPRM1 118G* allele have a blunted response to morphine compared to those with the *118 A/A* genotype (53, 54). The *OPRM1 A118G* polymorphism has also been associated with post-operative side effects such as vomiting (55). Additionally, intrathecal fentanyl injections are significantly more analgesic in women carrying the *OPRM1 304G* allele (56).

Gamma-aminobutyric acid (GABA) is the major inhibitory neurotransmitter in the mammalian brain and coordinates many physiological states, including sleep, anesthesia, and pain modulation (57). Barbiturates such as isoamylbarbital, pentobarbital, and secobarbital alter the activity of GABA-A and glycine receptors, inducing CNS depressant and sedative effects. Due to their tendency to cause respiratory depression in preoperative anesthesia, they have been replaced by benzodiazepines with safer pharmacological profiles (16, 17).

Midazolam and diazepam are orthosteric modulators of the GABA-A receptor. Choi et al. (58) found that patients with the *AA* genotype of *GABRA1* (the α-1 subunit of the GABA receptor) *rs4263535* have an increased risk of deep sedation. Malignant hyperthermia (MH) is a rare autosomal dominant disorder characterized by sudden onset of muscle spasms, rapid temperature increase, tachycardia, elevated heart rate, and an increased risk of heart failure, along with increased oxygen consumption, acidosis, and myoglobinuria. Mutations in the *RYR1* gene, which encodes the ryanodine receptor on the sarcoplasmic reticulum, are one of the possible causes (59). Volatile anesthetics and succinylcholine are the most common triggers of MH in susceptible patients. Testing all patients suspected of having MH can reduce MH mortality (60). Anesthetics and their adjuvants are critical in surgery, and this pharmacogenomic evidence underscores the potential for genetic analysis to inform anesthetic selection and dosing, with the aim of minimizing adverse effects and maximizing therapeutic outcomes.

Anesthesiologists can use this knowledge to understand the effects of anesthetics on perioperative disease. Additionally, opioids, ketamine, and non-steroidal anti-inflammatory drugs are used in treating chronic cancer pain. Studies have shown that perioperative administration of opioids that interact with specific tumor genomes can alter survival outcomes (61). Table 2 lists some factors associated with anesthesia for certain cancers, although causality has not been established. These factors could ultimately influence anesthesia and analgesia in cancer patients by considering changes in perioperative and pain management interventions for specific cancer subtypes (62). Incorporating these genetic factors into clinical practice may help to tailor anesthesia care to match the genetic makeup of each patient.

**TABLE 2:** Summary of drugs and tumor-related factors in anesthesiology.

Drug	Tumor-related proteins, genes, etc.	Cancer	Result
Volatile anesthetics	VEGF-A	Ovarian cancer	Isoflurane, sevoflurane and desflurane significantly increased the expression of VEGF-A, MMP-11, CXCR2 and TGF-β genes and enhanced the metastatic potential of ovarian cancer (63)
	MMP-11		
	CXCR2		
	TGF-β		
	VEGF	Renal cancer	Benzonana et al. (64) found that isoflurane affects VEGF and HIF in correlation with poor prognosis in renal cancer, while renal cancer cells exposed to isoflurane cultures exhibited greater migration
	HIF		
	CD39	Colorectal cancer	Oh et al. (65) found no difference in the expression levels of CD39 and CD73 in circulating regulatory T cells between propofol and sevoflurane anesthesia groups during colorectal cancer surgery. Many perioperative factors may influence perioperative immune status during colorectal cancer surgery, and the effect of anesthetics may be minimal
Propofol	CD73		
	GABA-A receptor	Breast cancer	Garib et al. (66) found an increase in the percentage of migrating cells after exposing breast cancer cells to isoproterenol The group also reported that isoproterenol increases migration of breast cancer cells through activation of the g-aminobutyric acid A (GABA-A) receptor
	HIF-1α	Non-small-cell lung cancer	Propofol reduces cancer cell invasiveness by decreasing HIF-1α upregulation (67)
	TGF-β-1	Osteosarcoma	Propofol reduces sarcoma cell proliferation and invasion and increases apoptosis by down-regulating transforming growth factor beta-1 (TGF-β-1) (68)
Opioid analgesics	CDKN2A	Lung adenocarcinoma	Next-generation sequencing in a cohort of lung adenocarcinoma patients undergoing primary tumor resection found that increased intraoperative opioid dosage was associated with poorer overall survival and that this association was amplified in patients with mutations in the *CDKN2A* tumor suppressor gene, the alterations in the Hippo and Wnt pathways, combined with increased doses of opioid drugs, are associated with a reduced recurrence rate of tumors (61)
	MMR-D	Colon adenocarcinoma	There is an association between higher intraoperative opioid doses and reduced recurrence rates, which is amplified in patients with specific tumor mutations in the DNA mismatch repair system (termed MMR trapped or MMR-D). These dMMR patients are characterized by an activated immune response in the tumor microenvironment, which can affect prognosis and response to immunotherapy (69, 70)
Non-steroidal anti-inflammatory drugs	NRF2	Lung adenocarcinoma	Alterations in the nuclear factor erythroid 2-related factor 2 (NRF2) oncogenic pathway or the *MDM2* gene (part of the TP53 oncogenic pathway) may reverse the association of ketorolac with poorer and improved recurrence-specific survival (71)
	MDM2		

## 3 Biomarkers

Biomarkers are not only key indicators of infection and host response dysregulation but also valuable tools for assessing treatment responses. They assist clinicians in predicting patient risks and serve as diagnostic and prognostic tools for clinical decision-making and risk stratification in clinical trials (72, 73). Beyond indicating systemic manifestations of infection and organ dysfunction, biomarkers offer insights into the biological basis of disease pathogenesis and treatment outcomes (73, 74). The detection of specific biomarkers during the perioperative period can enhance understanding of a patient’s condition, guide the development of tailored anesthetic regimens for various outcomes, and reduce patient risks while improving prognosis. For example, specific combinations of protein biomarkers can identify patients with adult respiratory distress syndrome (ARDS) who are most likely to benefit from interventions such as positive end-expiratory pressure (PEEP) or conservative fluid management strategies (75, 76).

Postoperative cognitive deficits are primarily categorized as postoperative delirium (POD) and postoperative cognitive dysfunction (POCD) (77, 78). Postoperative delirium (POD) is an acute and transient dysfunction of the central nervous system (CNS) (79) occurring in 15–53% of elderly patients immediately after surgery (80). Unlike delirium, POCD is not a clinical diagnosis but rather a variable operational concept, defined by postoperative cognitive decline as measured by neuropsychological testing within the first three months after surgery (80). POD and POCD are closely associated with neuroinflammation (81) and several biomarkers can predict or diagnose the occurrence of both (see Table 3). An increasing number of preclinical studies have shown that general anesthetics cause long-term cognitive impairment (82). A recent meta-analysis revealed that the use of midazolam, propofol, desflurane, and sevoflurane was associated with a higher incidence of delirium compared to dexmedetomidine (83). The anti-inflammatory and immunomodulatory effects of dexmedetomidine have been shown to reduce acute POD (84). Moreover, the use of benzodiazepines, opioids, antihistamines, and dihydropyridines has been linked to an increased risk of delirium (85). Several studies have indicated that isoproterenol, dexmedetomidine, and fentanyl reduce the risk of cognitive impairment compared to agents like midazolam, lorazepam, pethidine, and morphine (86). However, O’Bryan et al. (87) found statistically that the choice of maintenance anesthetic had little effect on the perioperative inflammatory response. Instead, individual patient and surgical factors may have a greater influence on the inflammatory response. The impact of anesthetic agents on postoperative cognitive impairment warrants further investigation, and monitoring relevant biomarkers could aid in risk stratification and improving prognosis.

**TABLE 3:** POD/POCD-related biomarkers.

Biomarker	Biomarker function	Result
IL-1β IL-6 TNF-α	Inflammatory	Surgery and anesthesia can induce upregulation of levels of CNS inflammatory factors such as IL-1β, IL-6 and TNF-α, causing central inflammation and consequently POCD (88)
IgM	Inflammatory	Low levels of IgM in the preoperative period may be one of the predictive markers of POCD, and increasing IgM levels or reducing the endotoxic inflammatory response improves cognitive dysfunction after cardiac surgery (89)
β-amyloid(Aβ)	Metabolite	Excessive deposition of Aβ is also an important mechanism for cognitive decline, and Aβ production and deposition can trigger POCD (90–92)
HMGB1	Inflammatory	When inflammation occurs, high expression of HMGB1 is observed in and around the hippocampus of the brain of experimental animals. Peripheral inflammatory signals were altered by elevated HMGB1 expression, which further caused CNS dysfunction and affected postoperative cognitive functions (93–95)
S-100β	Calcium-binding protein	S-100β protein is relevant in predicting POCD, and overexpression of S-100β protein further deteriorates the neuroinflammatory response and neuronal function (88)
MMP9	Inflammatory	Patients with POCD have elevated levels of the plasma inflammatory marker MMP9, and its correlation with POCD needs to be further investigated as a potential POCD-related biomarker (96)
NfL	Neuronal axonal damage	POD is associated with neuronal axonal damage, and increased NfL predicts POD, supporting that NfL can be used as a biomarker for POD (97, 98)
CRP	Inflammatory	CRP is one of the markers of central inflammation; however, there are limitations in using CRP as a marker of cognitive function due to its low disease specificity
Tau	Axon protein	Plasma Tau protein was significantly correlated with the incidence as well as the severity of POD (99)
pNF-H	Neuronal cytoskeletal proteins	Serum pNF-H, a new central damage marker highly correlated with POD, may reflect the occurrence and severity of POD (100)

POD, postoperative delirium; POCD, postoperative cognitive dysfunction; CNS, the diagnosis of central nervous system; IL-6, interleukin-6; IL-1β, interleukin 1β; TNF-α, tumor necrosis factor-α; IgM, immunoglobulin M; β-amyloid, amyloid β-protein, Aβ; HMGB1, high mobility group box-1 protein; S-100β, the soluble protein-100β; MMP9, matrix metalloprotease 9; NfL, neurofilament light chain; CRP, C-reactive protein; Tau, tau protein; pNF-H, phosphorylated neurofilament heavy chain.

## 4 The role of artificial intelligence (AI) and machine learning in personalized anesthesia

Artificial Intelligence (AI), which analyzes and classifies complex patterns and large amounts of data, is increasingly being recognized in healthcare for its ability to analyze complex datasets, simulate human cognitive learning, and incrementally improve its performance. Its applications range from virtual patient assistance to medical imaging and diagnostic support. Machine learning, a subset of artificial intelligence, has demonstrated the capability to assimilate clinical data to guide decision-making (101). In other clinical settings, image analysis is an area where AI approaches hold great promise (102). The wider application of AI in endoscopy could improve benign adenoma detection rates and reduce both the costs and risks of unnecessary polypectomies (103). AI-assisted image analysis aimed at improving disease risk prediction and diagnosis could detect cancer metastases (104), diabetic retinopathy (105), and identify benign melanomas (106). AI-based image analysis has also become part of direct-to-consumer diagnostic tools for anemia (107). In an attempt to automate the classification of pediatric pneumonia based on lung ultrasound patterns, neural network algorithms were able to correctly identify pneumonic infiltrates in healthy lungs with over 90% sensitivity and 100% specificity (108).

The emergence of diagnostic decision support tools has brought about a paradigm shift in anesthesia practice, combining human expertise with the computational power of artificial intelligence (AI) and machine learning (ML) (109). Decision aids aim to prepare individuals for decision-making by providing accurate and balanced information about treatment options and outcomes, helping them make specific and considered choices about their treatment (110). These aids have shown effectiveness in helping patients recognize the value sensitivity of decisions, guiding them to consider benefits and harms, improving patient-provider communication, and providing guidance throughout the decision-making process (110). Additionally, decision aids assist patients in making informed healthcare decisions by offering detailed information about treatment options and outcomes (110, 111). AI is utilized for evidence-based clinical decision support (112), detecting adverse events, and using electronic health record (EHR) data to predict patients at risk of readmission (113). By accessing EHR data, AI has demonstrated potential to surpass physicians in diagnostic accuracy (114–117). Algorithms that combine imaging and EHR data with relevant medical records can predict malignancy on biopsy and differentiate between normal and abnormal screening results, significantly reducing missed breast cancer diagnoses (118). AI-enabled clinical decision support systems can reduce diagnostic errors, enhance decision support intelligence, and assist clinicians with EHR data extraction and documentation tasks. Moreover, Banegas et al. (119) found that the use of decision aids reduced decisional conflict and aided women at high risk for breast cancer in deciding whether to take prophylactic tamoxifen or raloxifene to reduce cancer risk.

The development of artificial intelligence in all aspects of anesthesia has brought significant benefits, including airway management, ultrasound-guided interventions, intelligent drug infusion systems, accurate intraoperative monitoring, and perioperative risk assessment (120). A randomized trial evaluating the performance of an automated inspired oxygen concentration (FiO2 closed-loop system) using a narrower SpO2 target range found that the time spent within the clinically determined alarm limit (86–94%) was as good as with two wider target ranges (121). In an attempt to automate the classification of pediatric pneumonia based on lung ultrasound patterns, a neural network algorithm was able to correctly identify pneumonic infiltrates in healthy lungs with over 90% sensitivity and 100% specificity (108).

In genetic diagnostics, particularly for rare genetic diseases, clinicians face the daunting task of distinguishing disease-causing variants from millions of benign variants (122). Advances in artificial intelligence are transforming healthcare (123). and are expected to address bottlenecks in diagnosing rare genetic diseases through electronic clinical decision support systems (eCDSS) (124–128). A well-integrated CDSS linked to an electronic health record (EHR) can simplify data analysis and eliminate the need for redundant data entry.

Clinical validation and implementation of enhanced decision support tools are still in their infancy compared to other functionalities, and there is ample room for research on artificial intelligence and automation in anesthesia. The introduction of artificial intelligence and machine learning in medicine has already helped healthcare professionals improve the quality of care they provide and is expected to continue to do so in the near future and beyond. As these technologies advance, they offer a pathway for a more predictive and personalized approach to anesthesia, highlighting the need for anesthetists to become proficient in these digital tools to enhance patient care.

## 5 Artificial intelligence and real-time tools

### 5.1 Monitoring technologies: enhancing perioperative safety and efficacy

Advancements in monitoring technologies have significantly augmented the anesthesiologist’s ability to individualize patient care and optimize perioperative outcomes. Techniques such as electroencephalography (EEG) for assessing the depth of anesthesia and near-infrared spectroscopy (NIRS) for cerebral oximetry are at the forefront of these advancements.

The cerebral oxygen index (COx), correlating local brain tissue oximetry (StO2) derived from NIRS with mean arterial pressure (MAP), has become a pivotal tool in monitoring cerebral oxygenation. Tissue ischemia, hypoxia, hyperoxia, and hyperoxic reperfusion enhance the production of reactive oxygen species, thereby inducing oxidative damage (129–131). Such intraoperative oxidative stress has been implicated in postoperative cerebral and renal injuries (132). Cerebral oximetry, a non-invasive and user-friendly technique, allows for the real-time estimation of cerebral oxygen saturation (133). Recent findings by Lopez et al. (134) suggest that traditional practices of over-oxygenation during surgery, commonly believed to be protective, may in fact be deleterious to cerebral tissues.

Near-infrared spectroscopy has proven to be a reliable surrogate for cerebral blood flow, offering earlier warnings of compromised perfusion compared to traditional indicators of cerebral ischemia (135). Painful stimuli received by the CNS produce nociception (136). Localized cortical activation in adults not only results in nociceptive sensations but also causes an increase in local blood flow to the activated area (137) which significantly exceeds the oxygen demand of the brain tissue, ultimately leading to an increase in the oxygen content of hemoglobin. Functional near-infrared spectroscopic imaging, equivalent to magnetic resonance in assessing brain function, utilizes the distinct optical properties of hemoglobin to non-invasively quantify changes in cortical hemodynamics (138). Thanaboriboon et al. (138) demonstrated an increased risk of cerebral de-oxygenation events (CDEs) during shoulder arthroscopy in the beach chair position. The risk of CDE is high, and factors that may affect cerebral perfusion and oxygenation should be carefully monitored. Additionally, a study using near-infrared spectroscopy during shoulder arthroscopy in the beach chair position found that CDEs were more likely to occur (139).

Electroencephalography, historically used for diagnosing neurological diseases, now plays a critical role in monitoring the depth of anesthesia (140, 141). Frontal cortex EEG signals exhibit characteristic responses to anesthetic agents, leading to the development of various devices since the 1990s that utilize these EEG frequency domain transformations (142). Monitoring the depth of anesthesia (DoA) via EEG remains a challenge for anesthesiologists, especially in the elderly, due to age-related decreases in brain activity (143, 144), complicating the distinction between awake and anesthetized states in individual patients.

### 5.2 Enhancing anesthetic precision with AI-integrated monitoring technologies

The integration of artificial intelligence (AI) with monitoring technologies marks a significant advancement in anesthesiology, leveraging AI’s formidable data processing and self-learning capabilities. By statistically analyzing the continuous data streams from anesthesia machines and monitors, AI can harmonize with technologies like electroencephalography (EEG) and near-infrared spectroscopy (NIRS), providing real-time feedback on anesthetic depth to optimize patient care.

Machine learning models, built upon AI foundations, have demonstrated their utility in perioperative anesthesia management. These models enhance the interpretation of EEG signals, facilitating nuanced analyzes of complex data streams for depth of anesthesia (DoA) monitoring. Studies have highlighted the efficacy of direct EEG signal analysis through AI and spectral analysis (120). Park et al. (145) developed a DoA system utilizing real-time EEG and deep neural network algorithms that surpass traditional bispectral index (BIS) systems in performance. Gu et al. (146) devised a monitoring system integrating multi-electroencephalographic frequencies and entropy features with neural networks to classify DoA stages with remarkable accuracy. Ramaswamy et al. (147) extracted EEG spectral features using clinical trial datasets, logistic regression, support vector machines, and random forest models, accurately predicting the depth of sedation in patients. Similarly, Mirsadeghi et al. (148) and Shalbaf et al. (149) have demonstrated the superior accuracy of machine learning algorithms over BIS in analyzing EEG features across various anesthesia depths. AI’s application extends to perioperative ultrasound, aiding anesthesiologists in swiftly and accurately interpreting images, enhancing the precision of perioperative assessments, and streamlining result analysis (148, 149).

The application of artificial intelligence (AI) extends beyond monitoring anesthesia depth, fundamentally enhancing perioperative ultrasound imaging. AI’s ability to swiftly and accurately process ultrasound images promises to revolutionize anesthesiologists’ workflows by improving the precision of perioperative diagnostics and reducing the time required for assessment analysis (120). Hayasaka et al. (150) successfully used AI to predict difficult intubations, while Hetherington et al. (151) designed a neural network model that identifies anatomical landmarks with up to 95% accuracy. AI also assists anesthetists in analyzing complex ultrasound data; this capability facilitates the performance of technically demanding procedures, such as epidural punctures and tube placements, by automatically locating vertebral bodies and intervertebral spaces. Furthermore, AI aids in analyzing complex ultrasound data, with machine learning algorithms now capable of autonomously measuring cardiac ejection fraction and assessing cardiac function—delivering results that rival the accuracy of cardiologists and offer greater consistency than traditional ultrasound evaluations (152).

Moreover, AI’s predictive capabilities extend beyond diagnostics to the logistical aspects of surgery, including predicting surgical duration, identifying cancelations in high-risk procedures, and estimating post-anesthesia care unit stays. These advancements pave the way for more tailored anesthesia management, catering to the unique needs of each surgical procedure and patient profile (153).

## 6 Comorbidities and patient factors

**FIGURE 2:**
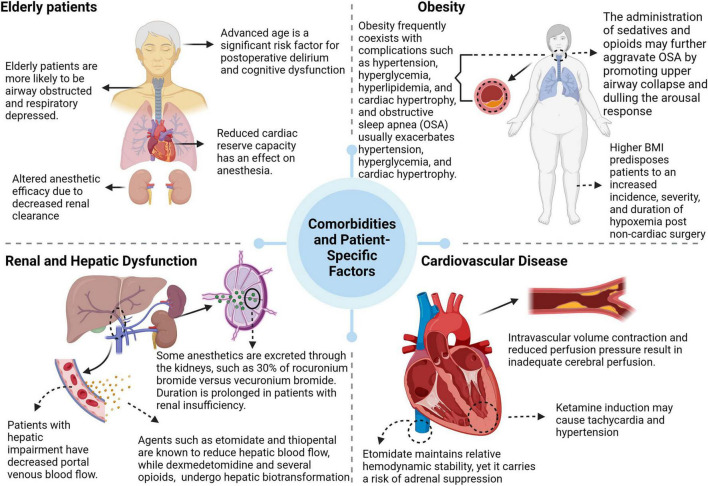
Age and obesity of patients or related complications affect the anesthetic effect.

An individual’s response to anesthesia is significantly influenced by various comorbidities and patient-specific factors (see Figure 2).

Age, particularly in the elderly, is an important determinant. This population has reduced cardiac, pulmonary, and renal reserve capacities and often exhibits physical and cognitive impairments. Anesthesia induces a complex physiological response in this group, which is exacerbated by the age-related decline in renal clearance. This decline prolongs the elimination half-life of both hydrophilic and lipophilic drugs, causing pharmacokinetic and pharmacodynamic changes that ultimately increase susceptibility to sedation (154). The clearance of benzodiazepines decreases significantly with age, enhancing their effects and increasing the risk of sedation-related adverse events (155, 156). Additionally, interactions between sedatives and centrally acting drugs, including general anesthetics, often produce synergistic effects (157).

Obesity is a multifactorial state of physiological dysfunction resulting from a complex interaction of genetic, environmental, and endocrine factors. It is often associated with comorbidities such as hypertension, hyperglycemia, hyperlipidemia, cardiac hypertrophy, and obstructive sleep apnea (OSA). The perioperative period in obese patients is compromised by an increased risk of pulmonary complications, typically characterized by altered respiratory mechanics, including increased respiratory rate, decreased tidal volume, and increased airway resistance (158, 159). Notably, obesity is a predictor of difficult airway management and is significantly correlated with difficult intubation scenarios (160).

This reduction in patients with hepatic dysfunction (especially those with cirrhosis) may be exacerbated by a lack of compensatory increase in portal blood flow during anesthesia (161). Drugs such as etomidate and sodium thiopental are known to reduce hepatic blood flow, whereas dexmedetomidine and several opioids (except remifentanil) undergo hepatic biotransformation and therefore require dose adjustment in the presence of hepatic insufficiency (162, 163). At the same time, renal disease alters the pharmacokinetics and pharmacodynamics of anesthetic agents. Rocuronium bromide and vecuronium bromide depend on renal excretion for 30% of their elimination and may have a prolonged duration in the presence of renal insufficiency. In contrast, cis-atracurium and atracurium, which undergo Hoffman elimination, are unaffected by renal impairment (164).

Cardiovascular disease poses a major challenge in the perioperative period, where surgical trauma, anesthesia, and related factors can induce arrhythmias, myocardial ischemia, and hemodynamic changes that can seriously affect patient prognosis (165). Patients with chronic hypertension tend to be more sensitive to anesthetics and surgical procedures (166).

The choice of anesthetic modalities and anesthetic drugs should be assessed in light of the patient’s condition and individual differences, and a personalized anesthetic plan should be developed based on the patient’s specific physiological conditions.

## 7 Challenges and future directions

### 7.1 Challenges in the implementation of personalized anesthesia

Despite its promise, personalized anesthesia still faces a number of significant obstacles. The limited availability and high cost associated with pharmacogenomic testing pose significant barriers to its widespread use. In the United States, clinical pharmacogenomic testing laboratories must be accredited by organizations such as the College of American Pathologists, and false positives and false negatives can occur due to potential errors in the test design itself (167). In addition, results are not standardized and may vary from lab to lab (168, 169). Second, the translation of pharmacogenomics into clinical practice requires the availability of high-quality genotyping tests in a short period of time, and the correct interpretation of pharmacogenetic test results by clinicians requires an adequate clinical decision support infrastructure, so it is necessary to train healthcare professionals (170) and to attempt to apply artificial intelligence and machine learning to create drug response prediction models to analyze genomic and other “histologic” data (171) to allow patients to choose the right drug at the right dose. As technology advances and costs decrease, genotyping may become more accessible and practical in routine anesthesia practice.

### 7.2 Navigating the pharmacogenomic landscape in anesthesia

The application of pharmacogenomic testing in clinical practice is compounded by the complexity of interpreting genetic data, particularly in the context of polypharmacy. Drug-drug interactions must be meticulously considered alongside pharmacogenomic results to accurately predict phenotypic outcomes (172). For instance, a patient concurrently taking multiple medications that prolong the QT interval (QTc) may be predisposed to torsades de pointes, even if pharmacogenomic testing predicts a normal response. Similarly, pharmacological agents that act as inhibitors or inducers can significantly alter the functionality of drug-metabolizing enzymes, thus transforming the phenotype (172). The integration of pharmacogenomic results with other clinical factors—such as age, existing comorbidities, and current medications—is imperative to avoid suboptimal patient outcomes (173). Any variant of a gene can affect the efficacy and safety of a drug, and 95.12% of all genes have one or more variants. Therefore, the detection of variants within key genes is important. For instance, the presence of 64 variants within the rosuvastatin gene raises concerns about the impact of these variants, which could range from negligible to the induction of severe myopathy, affecting a significant portion of prescriptions (174).

Given the intricacies of pharmacogenomic data, the development of intuitive tools and comprehensive guidelines is essential to aid anesthesiologists in interpreting and applying genetic information to patient care, thereby facilitating the delivery of truly personalized anesthetic management.

### 7.3 Advancing education and training in anesthesiology

The evolving field of anesthesiology is increasingly incorporating the principles of genetics, pharmacogenomics and personalized medicine. To ensure that these advances are translated into improved patient care, anesthetists need to be educated and trained in these disciplines, either through books or courses. They should be able to detect genetic polymorphisms and biomarker changes and determine individual conditions, as well as be skilled in the use of EEG, ultrasound and other artificial intelligence decision support tools. This is not only an extension of existing knowledge, but also necessary for the modern anesthetist to deal with the complexity of an individual’s genetic profile when administering anesthesia.

### 7.4 Future research directions in personalized anesthesia

Future research efforts in personalized anesthesia are expected to refine and expand the scope of patient-specific anesthesia management. Areas of focus should include:

Genetic and biomarker discovery: The identification of novel genetic determinants and biomarkers that can reliably predict an individual’s response to anesthetics and analgesics is critical. As more and more large biobanks or sample libraries are linked to genomic data, this provides an opportunity for future pharmacogenomic studies to query genetic polymorphisms more easily. In addition, anesthesia-related biomarkers are under-researched, and a large number of clinical trials are needed to identify the appropriate markers that can predict the risk of perioperative complications and allow for pre-emptive intervention.

Health economics: It is imperative to evaluate the cost-effectiveness of personalized anesthesia strategies. Research should aim to delineate the economic benefits, such as reductions in healthcare costs and improvements in patient outcomes, attributable to the adoption of personalized approaches.

Technological innovation: The development and rigorous validation of advanced monitoring technologies are critical. These innovations should be capable of supporting the customization of anesthetic management, aligning with the nuances of individual physiological responses.

Clinical trials: There is a need for extensive, multicenter clinical trials to conclusively ascertain the efficacy and safety of personalized anesthesia modalities across diverse patient demographics and surgical disciplines. Such trials will be instrumental in establishing evidence-based guidelines and protocols.

As the field progresses, it is crucial that research in these areas is conducted with methodological rigor and a multidisciplinary approach, integrating insights from genomics, pharmacology, bioinformatics, and clinical anesthesiology.

## 8 Conclusion

Personalized anesthesia and precision medicine represent a paradigm shift in the field of anesthesiology, with rapid advancements heralding a new era of enhanced patient care. The integration of individual genetic profiles, specific comorbid conditions, and unique patient characteristics with the burgeoning fields of pharmacogenomics and biomarker discovery has the potential to significantly refine anesthetic management. When coupled with the latest in monitoring technologies, these insights empower anesthesiologists to customize treatment plans to the distinct requirements of each patient.

The path toward fully realizing the promise of personalized anesthesia is lined with challenges, including the need for widespread education and training in the relevant fields of genetics and pharmacogenomics, as well as the development of cost-effective and accessible technologies. Moreover, interdisciplinary collaboration is vital for advancing research and translating these innovations into routine clinical practice. As we navigate these challenges, the collective efforts of anesthesiologists, geneticists, and other healthcare professionals will be paramount in harnessing the full potential of personalized anesthesia to optimize patient outcomes and elevate the standard of care.

## Author contributions

SZ: Conceptualization, Investigation, Writing – original draft. QQ: Conceptualization, Investigation, Writing – original draft. WX: Data curation, Methodology, Writing – original draft. SY: Methodology, Validation, Writing – original draft. MZ: Validation, Visualization, Writing – original draft. HT: Validation, Visualization, Writing – original draft. JP: Formal analysis, Funding acquisition, Supervision, Writing – review & editing. JH: Formal analysis, Funding acquisition, Supervision, Writing – review & editing.
